# Estimation of actual evapotranspiration and water requirements of strategic crops under different stresses

**DOI:** 10.1038/s41598-025-92481-z

**Published:** 2025-03-05

**Authors:** Hadi Ramezani Etedali, Faraz Gorginpaveh, Ahmad Elbeltagi, Maryam Abdollahzadeh, Brian Collins, Ali Salem

**Affiliations:** 1 Department of Water Sciences and Engineering, Imam Khomeini International University, Qazvin, Iran; 2 Center for Complex Hydrosystems Research, Department of Civil, Construction and Environmental Engineering, The University of Alabama, Tuscaloosa, AL USA; 3 Agricultural Engineering Department, Faculty of Agriculture, Mansoura University, Mansoura, 35516 Egypt; 4 Centre for Sustainable Agricultural Systems, University of Southern Queensland, Toowoomba, QLD 4350, Australia; 5 Civil Engineering Department, Faculty of Engineering, Minia University, Minia, 61111 Egypt; 6 Structural Diagnostics and Analysis Research Group, Faculty of Engineering and Information Technology, University of Pécs, Pécs, Hungary

**Keywords:** AquaCrop, Crop model, Actual evapotranspiration, Real water requirement, Qazvin plain, Environmental sciences, Hydrology

## Abstract

According to the importance of water conservation in water scarcity regions, estimating the exact amount of required water for crops under different stress conditions in irrigation networks is vital. One of the challenges in water management is estimating these stresses with crop models. AquaCrop is a robust model that can simulate the actual evapotranspiration and the water needs under different biophysical and management conditions. In this study, the actual evapotranspiration (Eta) and the irrigation requirement of wheat, barley, and maize are estimated by the AquaCrop model in the Qazvin province, and then compared with the results of the CropWat model. According to the results, the irrigation requirement for all three crops was significantly less than the CropWat estimation that were 184, 55.9, and 38.6 mm less water volume is needed for wheat, barley, and maize, respectively, showing using this model, the water efficiency will increase and the less amount of water can bring us the same amount of production. After that, for better comparison and assessment of the AquaCrop model, results were compared to the amount estimated by the Moghan plain and represented a higher amount of the actual evapotranspiration and the irrigation requirement because of different climate situations. These differences are mostly due to the AquaCrop model that is able to adjust itself under different stress conditions.

## Introduction

Iran is located in dry and semi-dry regions, which encounters severe water shortage problems. These problems will affect the economy, ecosystem functions, and people’s well-being^1–4^. Non-uniform distribution of precipitation, population growth and the need for food because of that has exacerbated these problems. As agriculture is the primary user of freshwater, which uses 85% of the global surface and groundwater consumption^5–7^, defining strategies in the planning and management of available water resources in the agriculture sector is a national and global priority^8,9^. Addressing these challenges requires the adoption of advanced modeling tools capable of simulating crop water requirements under diverse climatic and management conditions. The need for a practical decision-making under stress conditions to provide food security highlights the importance of accurate modeling of agricultural strategies.

Determining the potential evapotranspiration (ET) and actual evapotranspiration (ETa) is a critical step in tackling these challenges^10–12^. Several strong studies have been dedicated to estimating ET and Eta^13–15^. ETa is engaged for more than 60% of precipitation returning to the atmosphere^16^. It also consumes about 50% of the solar radiation, as latent heat flux, absorbed by the Earth’s surface^17^. As ETa is one of the major components of the global hydrologic cycle, quantifying it is fundamental. However, because of its complex interactions across the soil-vegetation-atmosphere interface, investigating ET_a_ is challenging^18–20^.

There have been numerous simulation methods in different ways that can increase the efficiency and performance of different systems, which cover a wide range of fields from infrastructure management to hydrometeorology^21–27^. In the past decades, several crop simulation models have been introduced that answer the concerns of stress on crops from beginning to yield (e.g., DSSAT^28^; CropSyst^29^; APSIM^30^; Hybrid-Maize^31^). However, most of these models’ use is limited due to their requirement for highly detailed input data about crop growth, which may not be available in some locations. FAO developed AquaCrop, a multi-crop model that simulates the herbaceous crop’s water-limited yield under different biophysical and management conditions with a good balance between robustness, simplicity, and output accuracy^32,33^.

Some studies used the AquaCrop model that simulates under water stress conditions are wheat^34–36^, quinoa^37^, potato^38,39^, barley^40^, corn^41^, cabbage^42^, cotton^43^, Bambara^44^, and Miscanthus^45^. In another study, the model’s probabilistic behavior has been studied and assessed with a Monte Carlo study^46^. It was also used in numerous studies in relation to the dataset and remote sensing techniques^12,47–49^.

Recently, Abdollahzadeh et al.^21^ estimated the actual evapotranspiration and the real water requirement of main cereals in the Moghan Plain with AquaCrop under real climate stress^21^. Generally, in water managing and water accounting, there might be this assumption that the crop is under no stress, and the following calculations depend on potential evapotranspiration. According to the literature review, there are few studies, according to the calibrated AquaCrop model by focusing on the available water requirement with actual evapotranspiration stresses. In this paper, the actual evapotranspiration and the actual water requirements of main crops (wheat, maize, and barley) in the Qazvin province will be estimated by the AquaCrop model. Estimations are evaluated and compared with the CropWat model. Finally, results will be compared to another region with different climate conditions. AquaCrop and CropWat models have emerged as widely used tools, each with unique strengths and limitations. AquaCrop is particularly suited for capturing stress conditions, while CropWat relies on simplified assumptions about ideal conditions, making a comparative evaluation of these models crucial for informed water management decisions. The objective of this study is to evaluate the performance of the AquaCrop model under stress factor under real field conditions. Also, we are interested in assessing the applicability of the model across different climatic conditions by analyzing two distinct agricultural regions with varying soil, precipitation, and temperature characteristiscs. In addition, we compared the results of the AquaCrop model against CropWat model. The results of this study will give us a better understanding of the precise water managemenet strategies in agriculture, especially in the regions with higher water scarcity. Also, we can see the impact of climateic scenarios with different soil conditions which allowing us for a broader understanding of te model’s applicability and reliability. Finally, this paper can show us how advanced modeling tools can support decision-making in water resources management over relatively large-scale regions.

## Materials and methods

### Case study

**Fig. 1:**
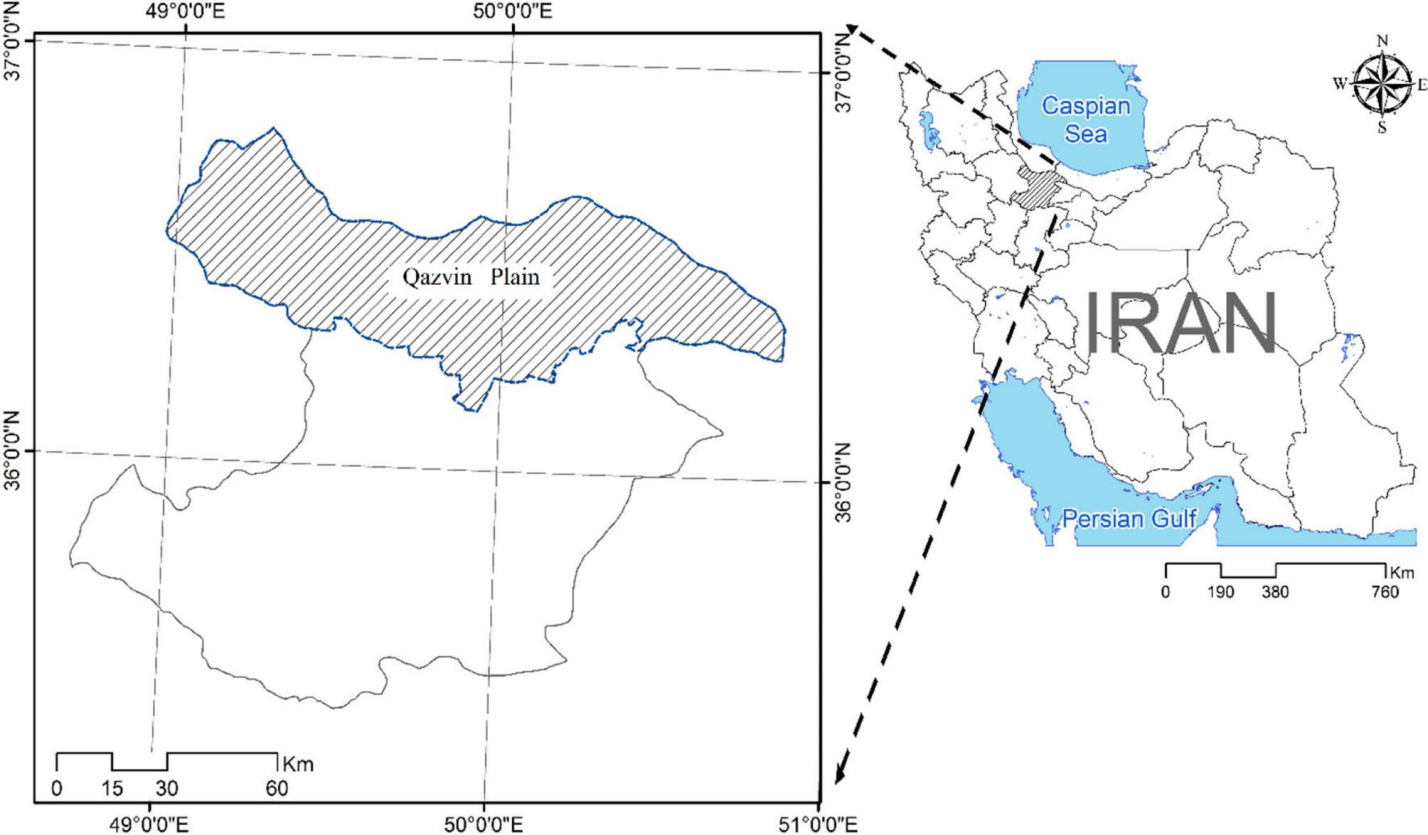
Qazvin plain in the Iran. The location of the Qazvin plain is in the north Qazvin Province. The girds of both the province and the plain are provided in the figure.

In this study, to estimate the actual evapotranspiration and compare results, two different regions were studied. The study region, the Qazvin Plain, with a 440 thousand ha area, is located in the central plateau of Iran. This plain is located in the Qazvin province, in 35 24’ to 36 48’ of north latitude from the equator and 48 44’ and 50 51’ of east longitude from Greenwich meridian (Fig. 1)^47,50^.

To compare the results of the actual evapotranspiration, another region, the Moghan Plain, was studied in this paper. Moghan, which is located in the Ardabil Province, was chosen because of its different climate conditions, and this can deepen our knowledge of the model under stress water and under different climates. This plain is located in the northwestern of Iran, in Ardebil province, with 300 to 350 thousand hectares. It is located between 47 35’ to 48 22’ of north latitude from the equator and 37 22’ to 39 45’ of east longitude from the Greenwich meridian.

**Fig. 2:**
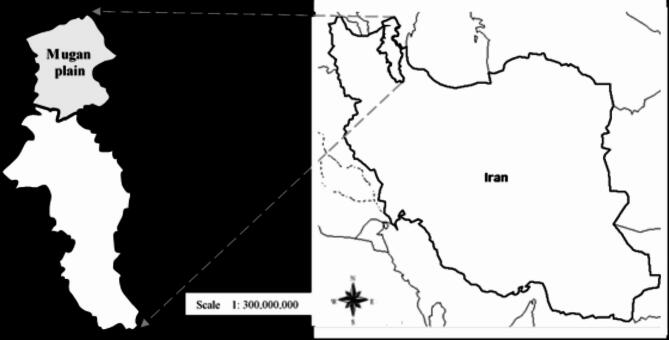
The location of Moghan Plain in Iran. As it can be seen, the Moghan plain is located in the north Ardebil province.

Both plains are crucial agricultural regions because of their suitable ecological conditions such as fertile soil, appropriate moisture, and temperature regimes. Wheat, barley, and maize are the main crops in these regions (Fig. 2).

### AquaCrop

In this study, the AquaCrop model version 5 is used to compute the actual evapotranspiration (ETa). Doorenbos and Kassam’s empirical relation (1980) simulates the yield and biomass of plants’ water^51,52^. The relation between crop yield and evapotranspiration in this model is as follows:

1 $$\left[ {\frac{{Y_{x} - Y_{a} }}{{Y_{x} }}} \right] = K_{y} \left[ {\frac{{ET_{x} - ET_{a} }}{{ET_{x} }}} \right]$$

which ET_x_ and ET_a_ are the maximum and the actual evapotranspiration, respectively, Y_x_ and Y_a_ are the maximum and actual yield, respectively, and Ky is the proportionality factor between relative yield loss and relative reduction ET. Furthermore, by separating evapotranspiration into plant transpiration and evaporation from the soil, this model can ignore the part of used water, which does not influence crop yield^53,54^.

Four categories of input data and the required data for each category are represented in (Table 1)^55^.

**Table 1:** AquaCrop inputs.

Soi	Management	Crop	Climate
Soil texture	Irrigation	constant values	Precipitation
Groundwater	Field	User specific parameters	Minimum temperature
			Maximum temperature
			Daily evapotranspiration of the reference plant (ET_0_)
			Carbon dioxide concentration

Although this mode is based on a complex bio-physical process, a relatively small number of simple and accessible parameters are used as input parameters.

The AquaCrop model was caliberated for both regions using historical field data, including crop growth stages, soil properties, and climatic variables such as temperature, precipitation, and evapotranspiration rates. Crop coefficients, soil hydraulic properties, and root zone depth were some parameters that was used for this study. Detail information of the field information are described below.

### Field and soil information

In this study, climate data of the Qazvin Plain was gathered from the years 1982 to 2013. In this plain, sprinkler irrigation with 0.5 dS/m was used. The calibrated information about the crops is shown in (Table 2).

**Table 2:** Plant parameters for wheat, barley, and maize.

Parameters	The number of days		
	Wheat	Barley	Maize
Germination	17	17	6
Flowering	175	175	66
Maximum vegetation	191	191	54
Start aging vegetation	201	201	107
Physiological maturity	235	235	132
Maximum depth of root development	97	97	108
Flowering period (day)	13	13	13
Maximum root depth (cm)	100	100	230
Primary vegetation (%)	3.37	3.13	0.49
Maximum vegetation (%)	80	80	90

For better comparison, the maximum and the minimum temperatures of both plains are represented monthly in (Table 3). In Table 4, the daily and monthly evapotranspiration and the amount of rainfall for both plains are represented. The FAO-Penman-Monteith equation estimated daily evapotranspiration. Precipitation values were gathered in the provinces’ meteorological station. Also, the effective precipitation of this plain is given in (Table 5). Finally, the information about the soil of these two plains is shown in (Table 6).

**Table 3:** Monthly maximum and minimum temperatures for the Qazvin and Moghan plains. The table highlights seasonal temperature variations, with Qazvin experiencing lower winter temperatures and Moghan showing higher summer peaks, reflecting distinct Climatic profiles.

Month	Qazvin plain		Moghan province	
	Maximum temperature	Minimum temperature	Maximum temperature	Minimum temperature
January	6.2	−4.1	8.9	−0.6
February	8.6	−2.5	9.8	0.7
March	14.1	1.4	14.9	4.1
April	20.6	6.6	19	8.2
May	26	10.3	26	13.8
June	32.6	14.8	31.5	18
July	35.4	17.6	33.9	20.9
August	34.9	17.1	33.4	20.5
September	30.8	13.2	28.3	17.3
October	23.4	8.3	21.8	12.1
November	14.6	3	14.9	5.8
December	8.3	−1.8	9.5	0.6

**Table 4:** Monthly precipitation and evapotranspiration (ET) data for the Qazvin and Moghan plains. The table illustrates the higher evapotranspiration rates in Qazvin during summer months and the comparatively consistent precipitation in Moghan, underscoring the impact of regional Climatic differences on water demand. (All values are in millimeters).

Month	Qazvin province			Moghan province		
	ET_daily_	ET_monthly_	Precipitation	ET_daily_	ET_monthly_	Precipitation
January	0.9	29.3	35.8	0.9	28.8	15.5
February	1.6	44.2	40.4	1.2	33	25.2
March	2.7	84.7	51.1	2	63.1	31
April	4	119.5	47.4	2.8	83.1	36
May	5.3	163.6	30.9	4.2	128.9	37.2
June	7.5	224.6	4.2	5.5	164.6	27
July	7.8	242.7	3.3	5.9	181.9	6.2
August	7.2	221.8	8.7	5.2	160.3	6.2
September	5.5	165.8	1.2	3.5	104.8	24
October	3.3	100.8	28.1	2	61.2	34.1
November	1.6	47.6	44.7	1.1	32.4	33
December	0.9	28.3	43.6	0.8	23.9	21.7
Year	–	1472.9	339.4	–	1066	297.1

**Table 5:** Average monthly effective rainfall of the Qazvin plain (mm).

Month	Rainfall (mm)
January	30.4
February	33
March	44.9
April	40.9
May	28.1
June	3.8
July	3.2
August	5.7
September	1.1
October	20.4
November	34.9
December	35.9

**Table 6:** Physical and soil hydraulic properties of Qazvin and Moghan plain.

Qazvin province				Moghan province			
Texture	FC (%)	PWP (%)	K (cm day^− 1^)	Texture	FC (%)	PWP (%)	K (cm h^− 1^)
Loam	32.2	16.1	25	Clay Loam	36.6	21.6	0.814

## Results and discussion

### Water requirement of the Qazvin plain

#### Water requirement Estimation of wheat

**Fig. 3:**
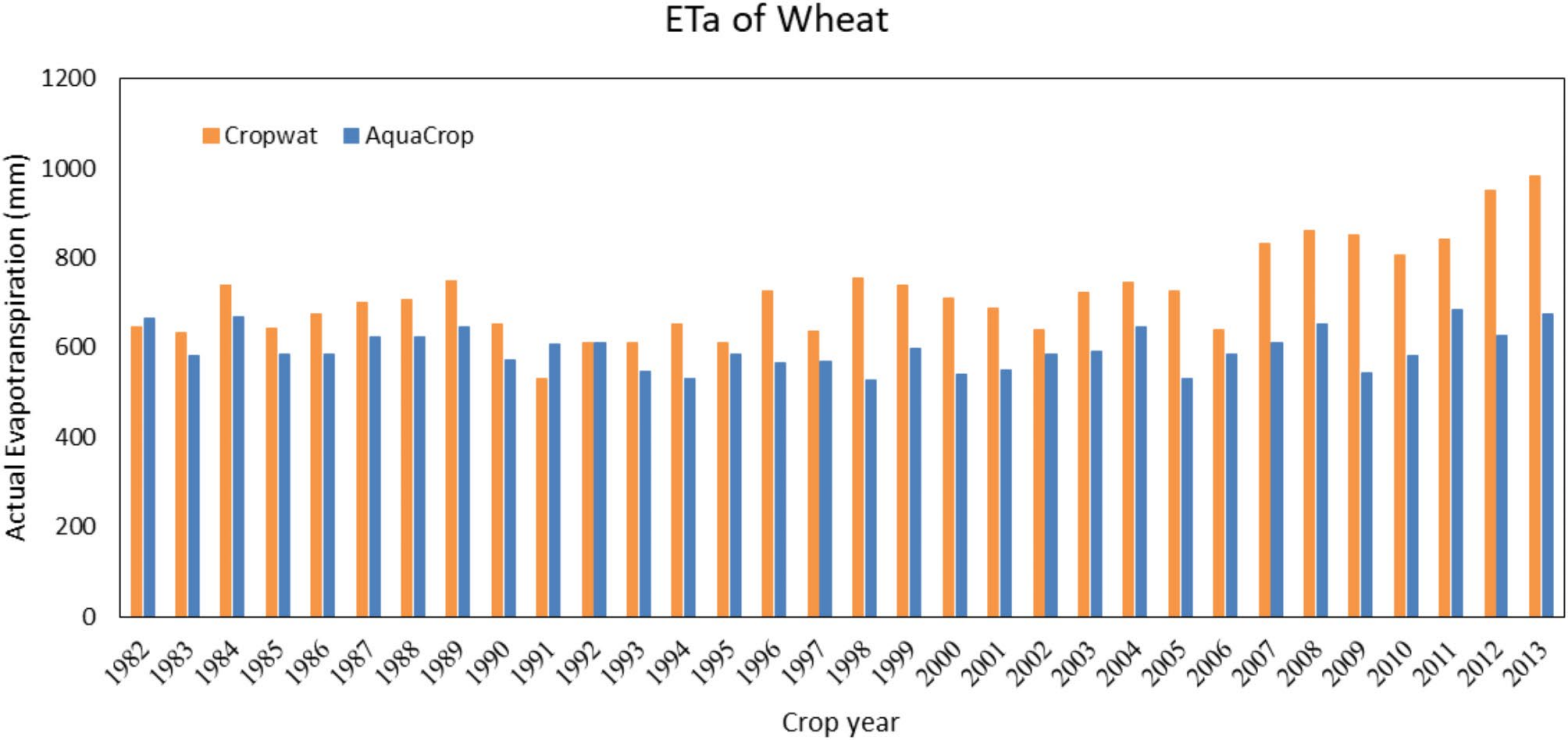
Actual seasonal evapotranspiration of Wheat in Qazvin based on calculations of the AqcuaCrop and the CropWat model. The higher values from the CropWat model are attributed to its inability to account for field stress conditions, resulting in an average overestimation of 136.8 mm per year compared to AquaCrop.

**Fig. 4:**
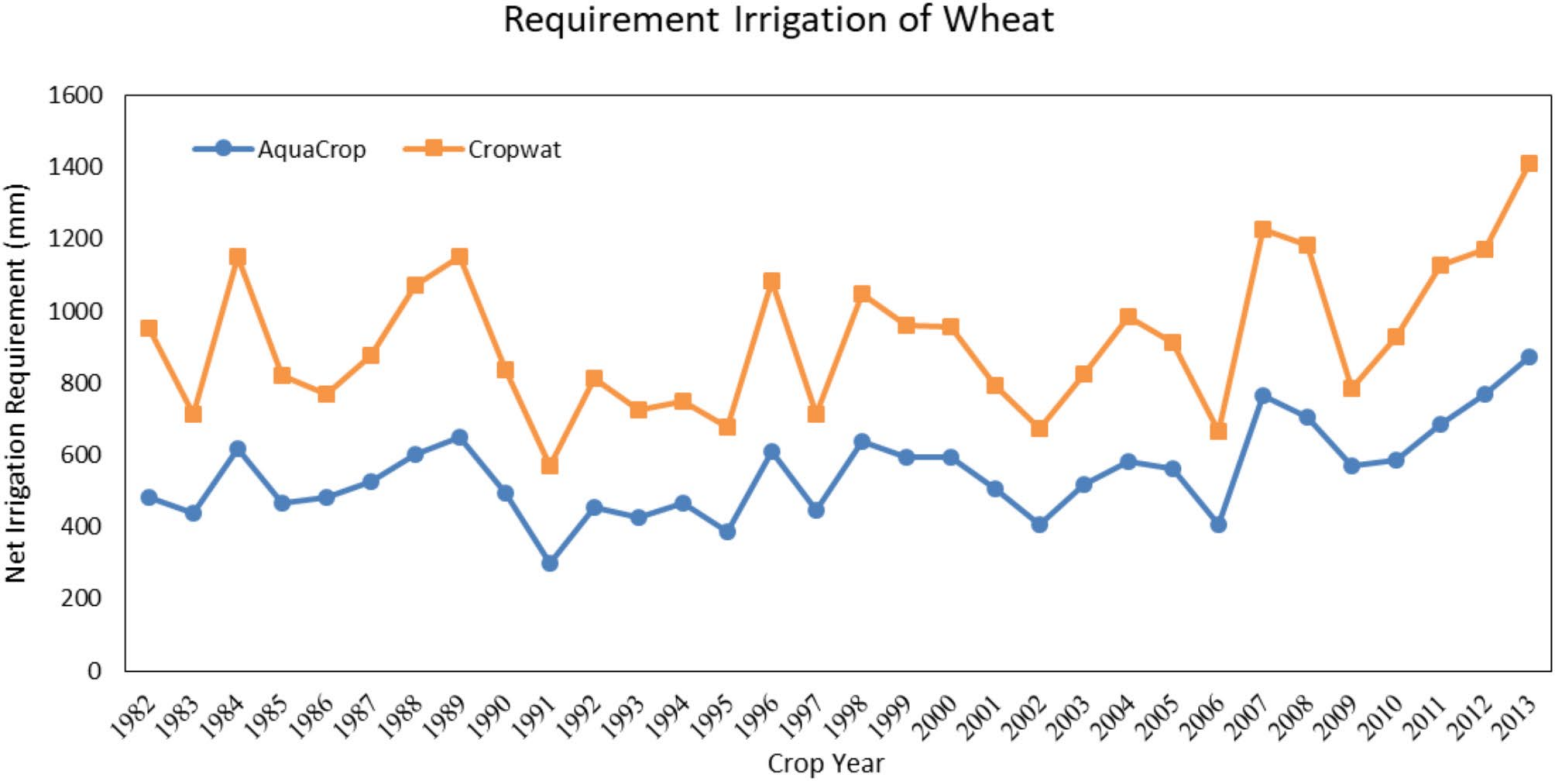
Seasonal wheat irrigation requirement in Qazvin based on calculations of the AquaCrop and the CropWat model. A significant reduction in irrigation needs predicted by AquaCrop due to its ability to incorporate water stress conditions.

The Irrigation planning (the number and the duration of irrigation) of the model was as same as the real amounts. The growth season of wheat in the Qazvin province starts on November 6th and continues to about July 6th the following year. According to Fig. 3, the actual evapotranspiration of wheat is estimated by the CropWat and the AquaCrop model. The reason for the higher amount of the CropWat estimation is neglecting the stresses in the field. This will lead to a 136.8 mm difference in estimating the real evapotranspiration each year. In Fig. 4, the amount of irrigation of wheat is estimated by the CropWat and the AquaCrop model. Similarly, the amount of estimation by the CropWat model is higher than the AquaCrop model due to the fact that the CropWat does not estimate the water stress. This amount is about 184 mm on average, which means that irrigation planning with the CropWat will need a higher water amount than the calibrated AquaCrop model.

#### Water requirement Estimation of barley

**Fig. 5:**
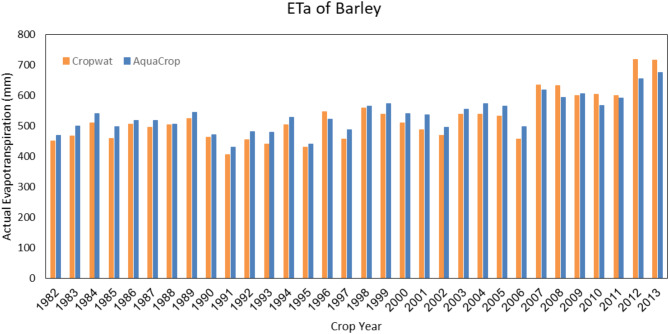
Seasonal barley irrigation requirement in Qazvin based on calculations of the AquaCrop and the CropWat model. Shwing the AquaCrop’s ability to optimize irrigation requirements by accounting for water stress conditions, resulting in reduced water usage compared to CropWat.

**Fig. 6:**
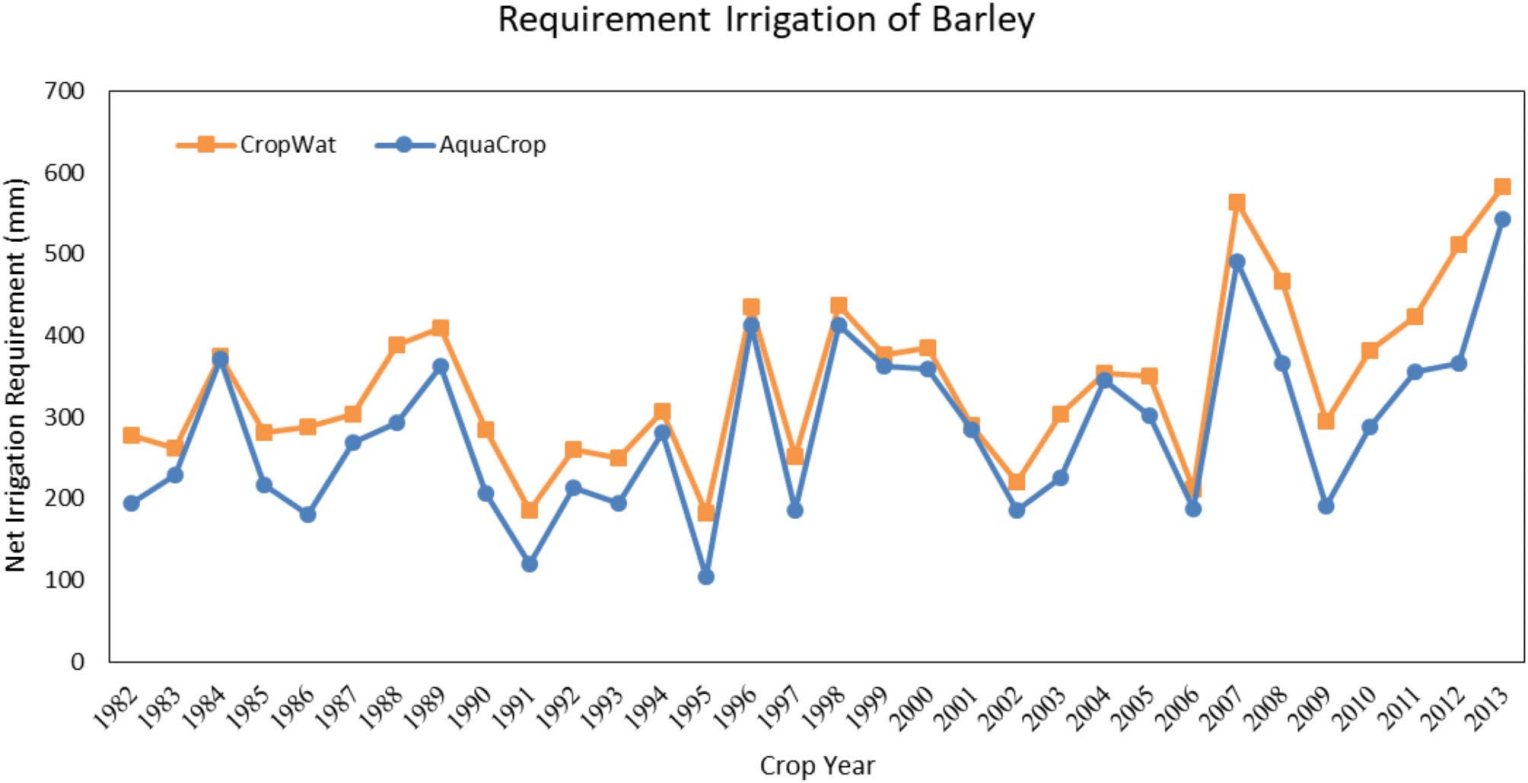
Seasonal barley irrigation requirement in Qazvin based on calculations of the AquaCrop and the CropWat model. The consistent reduction in water needs predicted by AquaCrop underscores its suitability for stress-inclusive irrigation planning.

The growth season of barley in the Qazvin province starts on October 23rd and finishes on June 20th. The actual evapotranspiration estimated by both models is represented in (Fig. 5). According to this figure, both models are estimated similarly. However, Fig. 6 shows that the estimation of irrigation requirement by the CropWat model is 55.9 mm higher than the AquaCrop model, averagely. These results show that irrigation planning with the CropWat model will need more water volume than the AquaCrop model.

#### Water requirement Estimation of maize

**Fig. 7:**
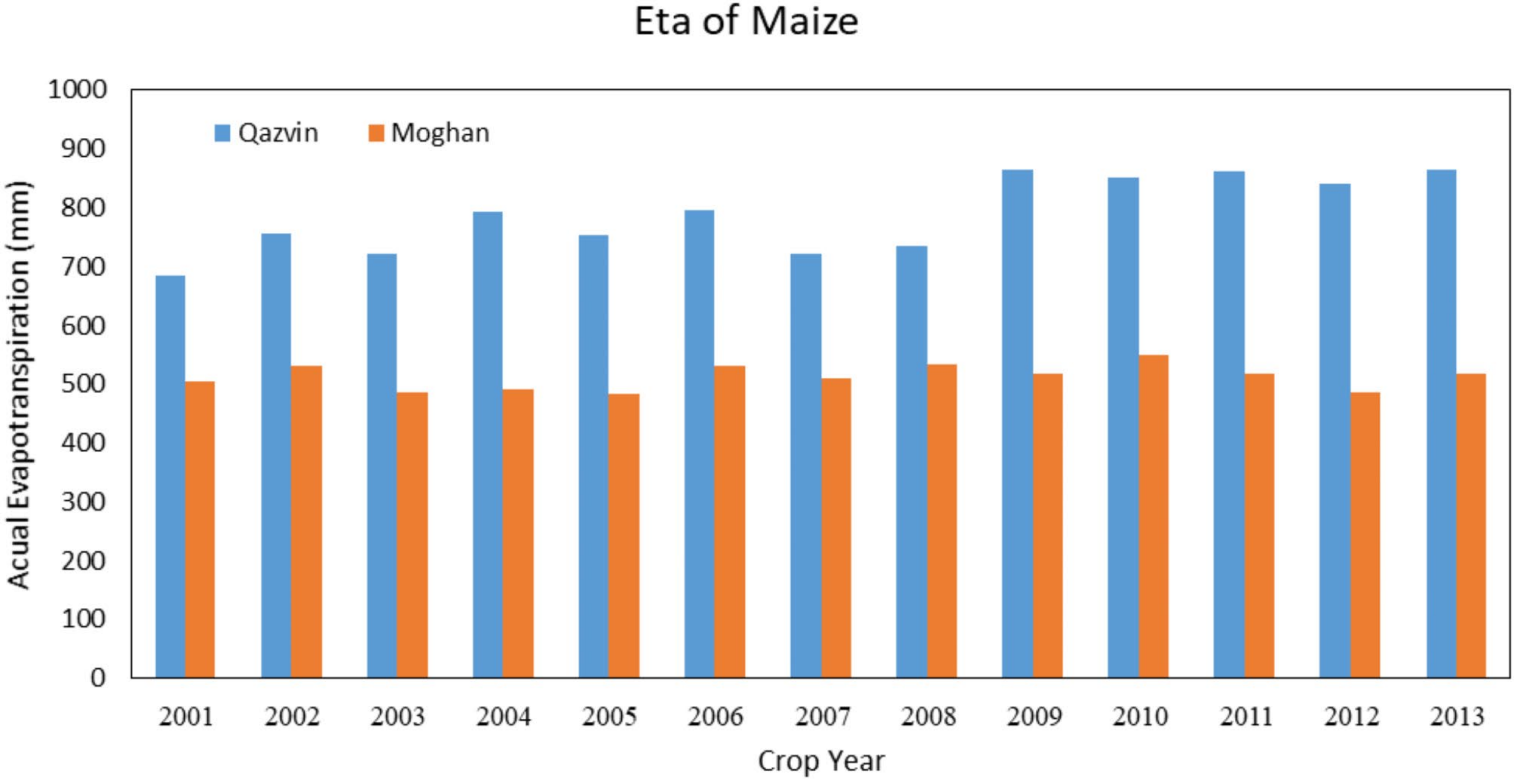
Seasonal maize irrigation requirement in Qazvin based on calculations of the AquaCrop and the CropWat model. Showing the AquaCrop’s ability to adjust irrigation needs based on year-to-year climatic variations, leading to more accurate predictions.

**Fig. 8:**
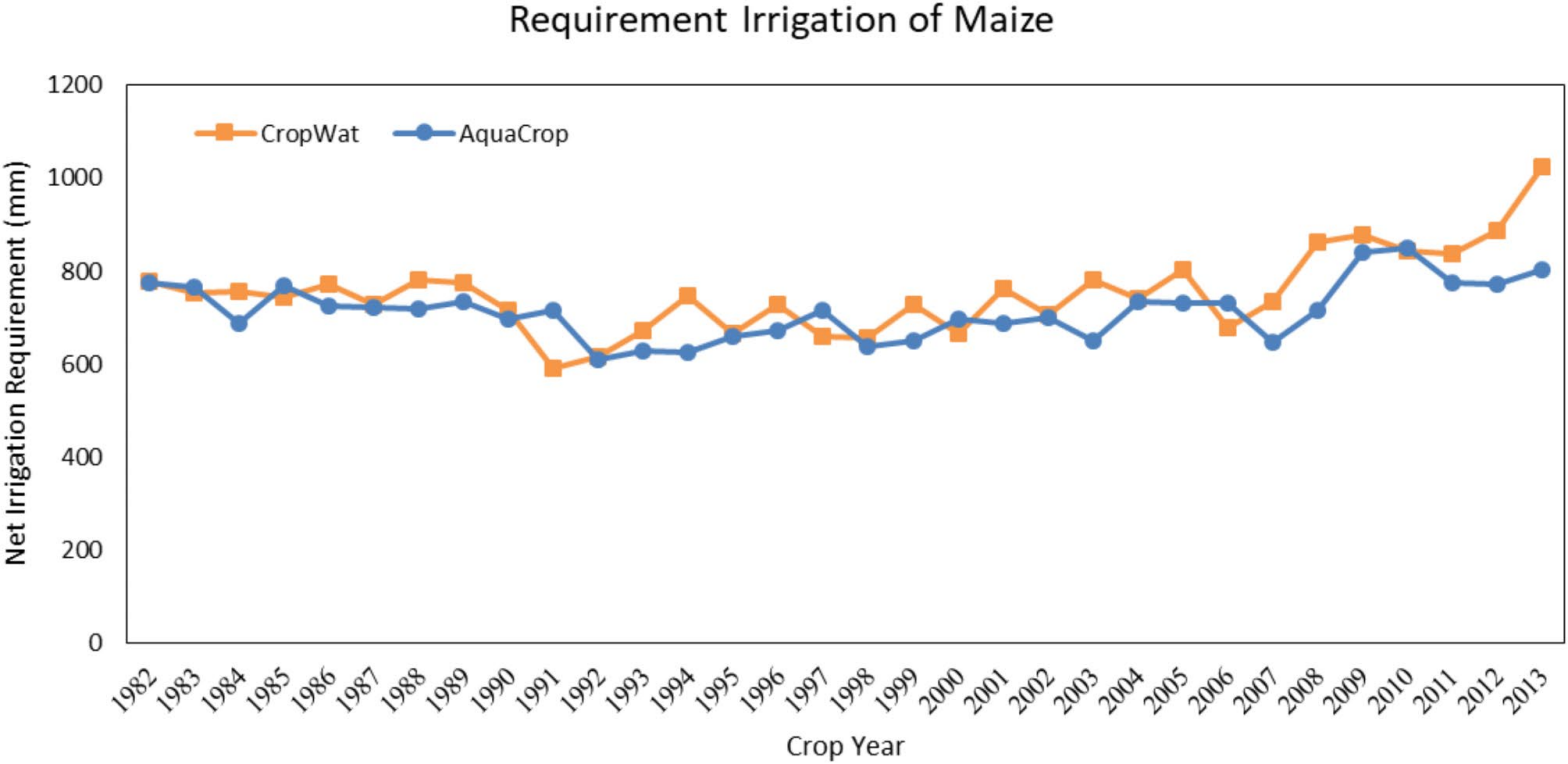
Seasonal maize irrigation requirement in Qazvin based on calculations of the AquaCrop and the CropWat model. The AquaCrop model’s lower irrigation predictions emphasize its efficiency in water management under stress conditions.

In the Qazvin province, the maize growing season starts on May 22nd and finishes on October 23rd. Figure 7 shows the actual evapotranspiration estimated by the CropWat and the AquaCrop model. As can be seen, both models estimate approximately similarly. This happens for crops that grow in summer since the climate fluctuations are less than the other seasons, and the irrigation will satisfy the crop’s water needs. The partial difference between the two models is due to the different temperatures each year for the AquaCrop, which is not considered by the CropWat model. According to Fig. 8 the irrigation requirement of barley in Qazvin, there is a difference between the two models (38.6 mm), which means the higher amount of irrigation needed by the CropWat model than the AquaCrop model.

According to the results, the evapotranspiration of crops is usually more than the actual evapotranspiration. The AquaCrop model, which estimates the actual evapotranspiration of crops’ under different stresses, estimates less amount of evapotranspiration. Also, the net irrigation requirement of the AquaCrop model shows less volume of irrigation water because of considering different stresses.

### Compare the results of both regions

**Fig. 9:**
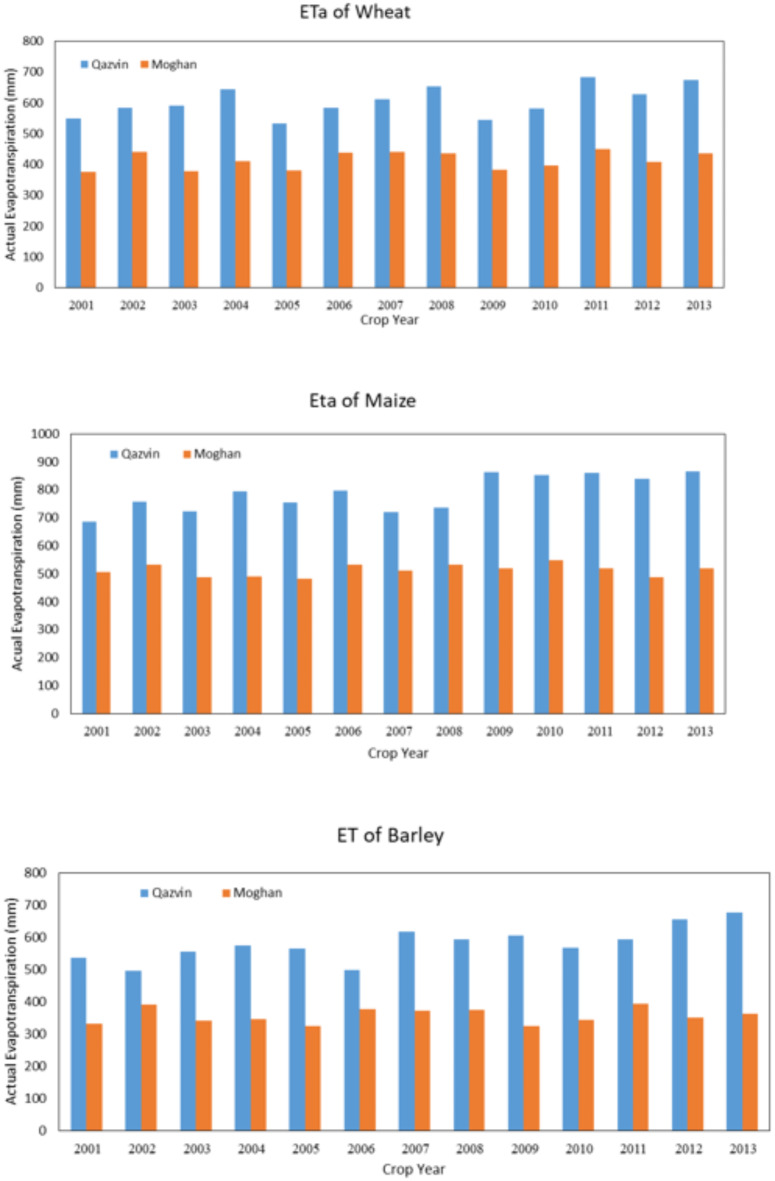
Actual evapotranspiration of wheat, maize, and barley in the Qazvin and Moghan Plains, as estimated by the AquaCrop model. The figure highlights variations in evapotranspiration due to differing climatic conditions between the two regions.

**Fig. 10:**
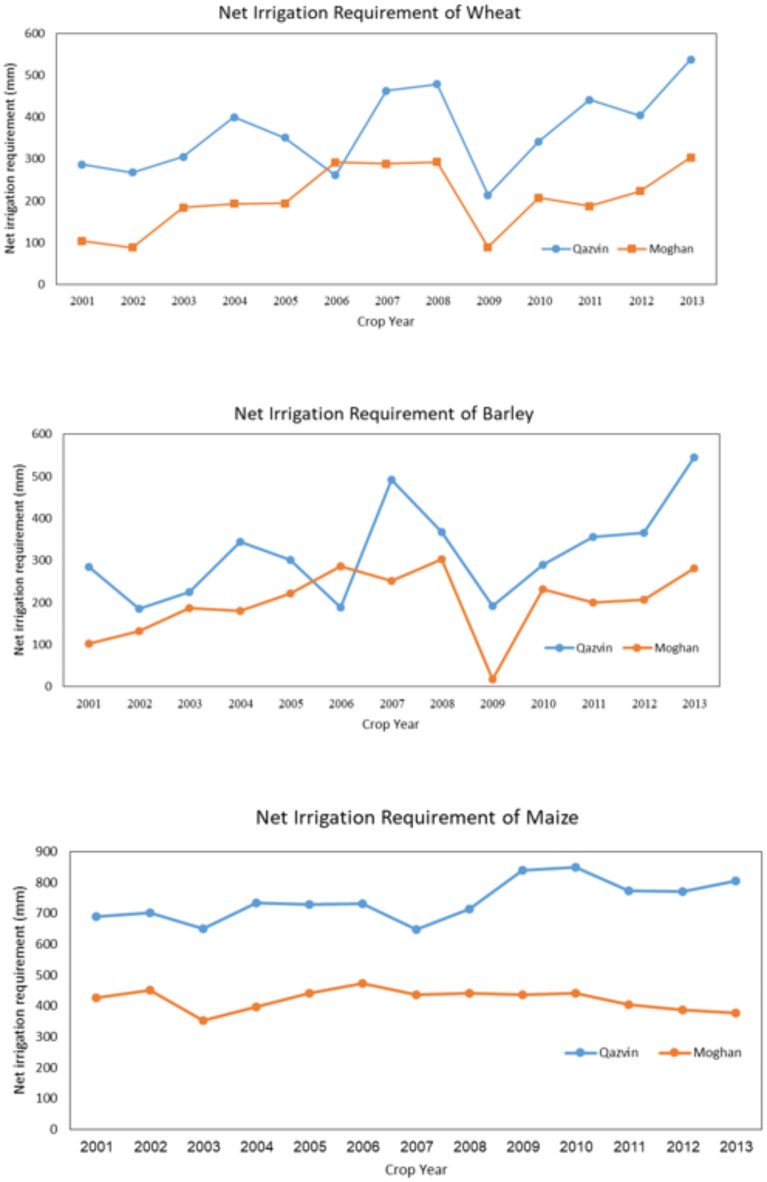
Comparison of irrigation requirements for wheat, maize, and barley in the Qazvin and Moghan plains using the AquaCrop model. Results emphasize the influence of regional climate variability on water requirements.

In Fig. 9, the results of the Qazvin plain in estimating the amount of actual evapotranspiration of wheat, maize, and barley are compared with the results of the Moghan plain for 13 years. According to this figure, in all three diagrams, the AquaCrop model’s amount is higher than the CropWat model. This is due to the different climate situations, such as different evapotranspiration, precipitation, and average maximum and minimum temperatures. The same difference in irrigation requirement is shown in (Fig. 10).

These results show the same behavior of actual evapotranspiration and net irrigation requirement of crops. They also confirm the high efficiency of AquaCrop in simulating accurately different regions with varying climates.

## Discussion

Some studies proved that the Kc values for the mild stage in cereals are mostly more than the development stage’s measured values^56,57^. Rushton et al.^58^ showed that irrigation water’s salinity would affect the amount of actual evapotranspiration based on the soil’s physical characteristics, soil moisture, and crop canopy^58^. Erkossa et al.^59^ investigated the effect of nitrogen fertilizer application on maize in Ethiopia. According to this study, the extra amount of nitrogen in the soil will increase the amount of transpiration up to 355 mm^59^. According to Zhong and Shangguan^60^, adding 270 kg.h-1 fertilizer to the soil will increase the total wheat evapotranspiration from 28, 14.1, and 23.1% in the three years of the study^60^. Toumi et al.^61^ considered the AquaCrop model as an operational tool for controlling the irrigation water of winter wheat in semi-arid regions^61^. Jin et al.^62^ concluded the AquaCrop model is a useful decision-making tool for optimizing wheat winter planting dates and irrigation strategies^62^.

Kumar et al.^57^ used the AquaCrop model to predict wheat yield and water productivity under irrigated saline regimes. They showed a better prediction of the model in grain yield compared to biomass and water productivity. Also, they claimed that the AquaCrop model needed less input data in simulating the wheat growth and yield under different saline irrigation availability scenarios^57^. Andarzian et al.^63^ used the AquaCrop model to estimate its efficiency under full and deficit irrigated wheat production. They showed that the model is able to simulate soil water content of root zone, crop biomass, and grain yield accurately with RMSE below 10%, with simplicity and minimum required input data^63^.

Farooq et al.^64^ studied the salinity stress in maize. They claimed that salinity stress reduces evapotranspiration, plant growth, photosynthesis, and plant organ formation^64^. Lacerda et al.^65^ showed that the maize evapotranspiration rates decrease by increasing the water salinity^65^. Abedinpour^66^ studied water use and wheat yield under different salinity irrigation water in Kashmar, Iran. He showed that the amount of daily evapotranspiration of wheat under salinity stress is lower than under no salinity stress^66^. Paredes et al.^67^ assessed AquaCrop in estimating maize and irrigation usage in full and deficit irrigation management. The model was assessed as an efficient model with RMSE lower than 11 and 9% of the average observed biomass and yield^67^.

Saeidi et al.^69–71^ assessed water salinity and deficiency of nitrogen in maize in Qazvin in several studies and claimed that the salinity water stress and the soil nitrogen could increase the Ks coefficient and reduce the crop evapotranspiration. Also, by estimating the real evapotranspiration of crops under stress treatment, water use management efficiency could increase. They claimed that adjusting the field water to the plant’s actual needs would prevent excessive consumption under salinity stress. They investigated that under water salinity and nitrogen stress, the water efficiency can be reduced up to 38%, and by reducing water use, water resources will be used optimally, and yield will increase. Finally, they proved that the accurate estimation of ET, which is reduced due to salinity, will result in a more accurate irrigation schedule and reduce the water footprint^68–71^.

Farahani et al.^72^ evaluated the AquaCrop model for full and deficit (40, 60, and 80%) cotton irrigation. The observed data tested the results of the modeling simulation. They showed the model simulation’s accuracy in predicting the total soil water trends^72^. Katerji et al.^73^ used AquaCrop for corn and tomato under water stress conditions. They showed the model could be considered reliable if the level of water stress, water stress coefficients, and simple corrections of the ETo or Kc values are correctly considered^73^. Linker et al.^74^ used the AquaCrop model to develop an optimization scheme for irrigation schedules of cotton, potato, and tomato. They showed that nonlinear constrained optimization could be used together with the model, achieving the highest yield achievable. Also, they deduced that this model could be adapted for other crops^74^.

Hellal et al.^75^ studied barley water efficiency at water deficit conditions. They assessed AquaCrop by the observed results in the field and claimed that the model was efficient in predicting the water unit’s productivity under semi-arid areas, especially in places facing water shortages^75^.

## Conclusions

Most irrigation planning is designed under no-stress conditions, which leads to an inaccurate amount of irrigation requirement. Most crop models cannot survey different stresses such as salinity, drought, high amount of temperature, fertility, and the depth of the soil. The AquaCrop model can compute the actual evapotranspiration due to considering different stress situations. In this study, the actual evapotranspiration and the irrigation requirement are estimated with the AquaCrop model for wheat, maize, and barley in the Qazvin province. Results were compared to the amounts estimated by the CropWat model. Results showed that irrigation planning with the AquaCrop model is more efficient than the CropWat model due to less need for water volume. In other words, irrigation planning with the AquaCrop model will reduce the amount of water usage of wheat, barley, and maize by 184 mm, 55.9 mm, and 38.6 mm on average. The results were similar to the literature review. Finally, this province’s results were evaluated with the results of another study in another region, the Moghan Plain. For all three crops, the behavior of estimating the actual evapotranspiration and the irrigation requirement in the Qazvin plain was similar and higher than the same amounts for the Moghan plain. This means the validity of using the AquaCrop model in different conditions and regions. These findings highlight the potential of the AquaCrop model to serve as a decision-support tool for policymakers and water resource managers. By integrating AquaCrop’s stress-sensitive irrigation estimates, regional irrigation policies can prioritize water-efficient practices tailored to specific climatic and crop conditions. This approach not only conserves water resources but also ensures sustainable agricultural productivity in water-scarce regions. Furthermore, adopting AquaCrop in regional planning can guide investments in irrigation infrastructure and inform strategies for climate resilience in agriculture.

## Data Availability

All data generated or analysed during this study are included in this published article.
